# The effects of antenatal dietary and lifestyle advice for women who are overweight or obese on maternal diet and physical activity: the LIMIT randomised trial

**DOI:** 10.1186/s12916-014-0161-y

**Published:** 2014-10-13

**Authors:** Jodie M Dodd, Courtney Cramp, Zhixian Sui, Lisa N Yelland, Andrea R Deussen, Rosalie M Grivell, Lisa J Moran, Caroline A Crowther, Deborah Turnbull, Andrew J McPhee, Gary Wittert, Julie A Owens, Jeffrey S Robinson, For the LIMIT Randomised Trial Group

**Affiliations:** School of Paediatrics and Reproductive Health, and The Robinson Research Institute, The University of Adelaide, Adelaide, Australia; Department of Perinatal Medicine, Women’s and Babies Division, The Women’s and Children’s Hospital, North Adelaide, Australia; Women’s and Children’s Health Research Institute, North Adelaide, Australia; School of Population Health, The University of Adelaide, Adelaide, Australia; School of Psychology, The University of Adelaide, Adelaide, AUSTRALIA; Department of Neonatal Medicine, Women’s and Babies Division, The Women’s and Children’s Hospital, North Adelaide, Australia; School of Medicine, The University of Adelaide, Adelaide, Australia; Liggins Institute, The University of Auckland, Auckland, New Zealand

**Keywords:** Pregnancy, Overweight and obesity, Diet composition, Physical activity, Randomised trial, Dietary and lifestyle intervention

## Abstract

**Background:**

Overweight and obesity is a significant health concern during pregnancy. Our aim was to investigate the effect of providing antenatal dietary and lifestyle advice to women who are overweight or obese on components of maternal diet and physical activity.

**Methods:**

We conducted a randomised controlled trial, in which pregnant women with a body mass index ≥25 kg/m^2^, and singleton gestation between 10^+0^ to 20^+0^ weeks were recruited and randomised to Lifestyle Advice (involving a comprehensive dietary and lifestyle intervention over their pregnancy) or Standard Care. Within the intervention group, we conducted a nested randomised trial in which a subgroup of women were further randomised to receive access to supervised group walking sessions in addition to the standard information presented during the intervention contacts (the Walking group) or standard information only.

The outcome measures were maternal dietary intake, (including food groups, macronutrient and micronutrient intake, diet quality (using the Healthy Eating Index; HEI), dietary glycaemic load, and glycaemic index) and maternal physical activity. Women completed the Harvard Semi-Structured Food Frequency Questionnaire, and the Short Questionnaire to Assess Health-enhancing Physical Activity (SQUASH), at trial entry, 28 and 36 weeks’ gestational age, and 4 months postpartum.

Analyses were performed on an intention-to-treat basis, using linear mixed effects models with adjustment for the stratification variables.

**Results:**

Women randomised to Lifestyle Advice demonstrated a statistically significant increase in the number of servings of fruit and vegetables consumed per day, as well as increased consumption of fibre, and reduced percentage energy intake from saturated fats (*P* < 0.05 for all). Maternal HEI was significantly improved at both 28 (73.35 ± 6.62 versus 71.86 ± 7.01; adjusted difference in means 1.58; 95% CI 0.89 to 2.27; *P* < 0.0001) and 36 (72.95 ± 6.82 versus 71.17 ± 7.69; adjusted difference in means 1.77; 95% CI 1.01 to 2.53; *P* < 0.0001) weeks. There were no differences in dietary glycaemic index or glycaemic load. Women randomised to Lifestyle Advice also demonstrated greater total physical activity (adjusted difference in means 359.76 metabolic equivalent task units (MET) minutes/week; 95% CI 74.87 to 644.65; *P* = 0.01) compared with women receiving Standard Care. The supervised walking group was poorly utilised.

**Conclusions:**

For women who are overweight or obese, antenatal lifestyle advice improves maternal diet and physical activity during pregnancy.

Please see related articles: http://www.biomedcentral.com/1741-7015/12/163 and http://www.biomedcentral.com/1741-7015/12/201.

**Trial registration:**

Australian and New Zealand Clinical Trials Registry (ACTRN12607000161426)

**Electronic supplementary material:**

The online version of this article (doi:10.1186/s12916-014-0161-y) contains supplementary material, which is available to authorized users.

## Background

Obesity is recognised as a significant global health problem [1], with well-documented risks for pregnant women and their infants, which rise with increasing maternal body mass index (BMI) [2,3]. Current clinical care guidelines indicate that ideally, women should be counselled prior to conception about the increased pregnancy risks associated with obesity, and encouraged to make lifestyle changes to reduce the risk of subsequent complications during pregnancy and childbirth through weight loss [4,5]. However, with data from Australia, the USA and UK suggesting more than 50% of women enter pregnancy with a BMI in excess of 25 kg/m^2^ [4,6,7], considerable attention has been directed towards evaluating interventions to limit gestational weight gain, and their effect on maternal and infant health outcomes [8-10]. Research has focused on the effects of dietary and lifestyle interventions to limit gestational weight gain, particularly among women who are overweight or obese [8-10], and there is limited information to date relating specifically to the effective components of these antenatal interventions, with suggestions that interventions designed to limit gestational weight gain through modification of diet may be more effective than those designed to increase physical activity alone [10].

Dietary quality can be assessed by utilising a number of different tools, including principal component analysis and use of dietary quality indices comparing nutritional intake with recommendations for healthy eating or dietary guidelines. A decrease in dietary quality, when utilising these measures as a comprehensive indicator of dietary intake, is associated with increased weight gain over time [11], as well as increased all-cause mortality and morbidity risk, specifically in relation to cardiovascular disease and some malignancies [12]. Compared with women of normal BMI, women who are overweight or obese demonstrate poorer diet quality during pregnancy [13], which continues into the early postpartum period [14]. When specifically compared with women of healthy weight, pregnant women who are overweight or obese demonstrate reduced intake of grains, vegetables, iron and folate [13,15,16]. With regard to clinical outcomes, poor diet quality during pregnancy has been associated with an increased risk of adverse pregnancy outcomes, including glucose intolerance and pre-eclampsia [17].

Dietary glycaemic index (GI) was developed as a tool for individuals with diabetes [18], ranking the post-prandial glycaemic response to ingested carbohydrates against a reference of either pure glucose or white bread, and the glycaemic load (GL) is the product of the glycaemic index and the amount of dietary carbohydrate [19]. Consumption of foods with low glycaemic index have been shown to be associated with reduced carbohydrate-induced post-prandial glycaemia [20]. Furthermore, a low glycaemic index or glycaemic load diet has been associated with improved weight loss through potential effects on hunger and energy intake [21]. Of relevance to pregnant women is the effect of maternal glycaemia on the placental transfer of glucose to the fetus, acting as a substrate for growth and development. While some studies have identified associations between maternal consumption of a low glycaemic index or glycaemic load diet and lower gestational weight gain [22,23] or lower infant birth weight [24], these findings are not universal, with other studies demonstrating no effect on infant birth weight [23,25-27].

Exercise during pregnancy is considered beneficial, improving maternal wellbeing and cardiovascular performance [28,29]. More specifically, exercise in pregnancy has been associated with a reduction in the risk of gestational diabetes [30,31], pre-eclampsia [32], and operative birth [33], and with improvements in fetal growth [34,35]. The American College of Obstetricians and Gynecologists (ACOG) has advocated that all pregnant women, without contraindications to exercising, should be active and participate in mild-to-moderate exercise for at least 30 minutes on most days of the week [36]. However, the recommendations for women who have been previously inactive or who have pregnancy complications are less clear-cut, requiring individual consideration [36]. ACOG recommend that pregnant women who are overweight or obese should be encouraged to follow an exercise programme in order to optimise health outcomes for both the woman and her infant [37]. Despite the proposed benefits of exercise, physical activity has been reported to decline over the course of pregnancy among women of all BMI categories, but is particularly evident among women who are overweight or obese [38-40], with a large proportion of women not attaining the recommended amount of exercise.

The primary findings of the LIMIT randomised trial evaluating the provision of antenatal dietary and lifestyle advice to women who were overweight or obese, have been reported previously, and indicate a significant 18% relative risk reduction in the chance of an infant being born with birth weight above 4 kg [41]. We now report the effect of providing antenatal dietary and lifestyle advice on specific components of maternal diet, including food groups, macronutrient and micronutrient consumption, dietary quality, and dietary glycaemic load and index, and maternal physical activity.

## Methods

### Ethics

Ethics approval was granted by the Women’s and Children’s Local Health Network Human Research and Ethics Committee at the Women’s and Children’s Hospital, the Central Northern Adelaide Health Service Ethics of Human Research Committee (Lyell McEwin Hospital) and the Flinders Clinical Research Ethics Committee (Flinders Medical Centre). All participants provided written informed consent.

### Study design

The study was a multicentre randomised trial across the three major metropolitan maternity hospitals within Adelaide, South Australia. The methods [42] and primary findings [41] of the LIMIT randomised trial have been reported previously, and the trial has been registered on the Australian and New Zealand Clinical Trials Registry (ACTRN12607000161426). Additional dietary and physical activity outcomes were added to the final working protocol, reflecting piloting and feasibility assessment, and finalization of specific questionnaires. These amendments were pre-specified in the final working protocol, early in the conduct of the trial, and prior to any analyses being undertaken.

### Inclusion and exclusion criteria

Women with a BMI ≥25 kg/m^2^ and singleton pregnancy between 10^+0^ and 20^+0^ weeks gestation were eligible for enrolment. Women with a multiple pregnancy, or with type 1 or 2 diabetes diagnosed prior to pregnancy, or who were unable to provide informed consent were ineligible.

### Trial entry

All women presenting for antenatal care had their height and weight measured and their BMI calculated at the first antenatal appointment. Eligible women were presented with written information, and were encouraged to discuss participation with their primary support person.

### Randomisation, masking and group allocation

Randomisation occurred by telephoning the central randomisation service, which utilised a computer-generated schedule, with balanced variable blocks. Stratification occurred for parity (0 versus ≥1), BMI at antenatal booking (25 to 29.9 kg/m^2^ versus ≥30 kg/m^2^), and collaborating centre. Women were randomised and allocated to either ‘Lifestyle Advice’ or ‘Standard Care’.

#### Nested randomised trial

Between January 2010 and September 2011, we conducted a nested randomised trial, in which women who were randomised to the Lifestyle Advice group underwent further randomisation to receive either written and verbal information about physical activity (Lifestyle Advice Group), or to additionally be invited to participate in a targeted, supervised walking group (Walking group).

### Treatment schedules

#### Lifestyle Advice group

Women randomised to receive Lifestyle Advice participated in a comprehensive dietary and lifestyle intervention over the course of their pregnancy, which included a combination of dietary, physical activity and behavioural strategies, delivered by a research dietician and trained research assistants [42]. Within 2 weeks of randomisation, the women attended a planning session with a research dietician, during which a detailed dietary and physical activity history was obtained.

Women were provided with dietary advice consistent with current Australian standards [43]: to maintain a balance of carbohydrates, fat and protein; to reduce intake of foods high in refined carbohydrates and saturated fats, while increasing intake of fibre; and to promote consumption of two servings of fruit, five servings of vegetables, and three servings of dairy each day [43]. Information was individualised, and included meal plans, healthy recipes that were quick to prepare, simple food substitutions (including reducing intake of sugar-sweetened soft drinks and fruit juices, reducing added sugar and foods high in refined carbohydrates, and using low-fat alternatives), healthy snack and eating-out options, and guidelines for healthy food preparation.

Physical activity advice focused on the benefits of exercise in pregnancy, potential safety concerns relating to exercise during pregnancy, tips to increase incidental activity and walking, and promoting recreational, aerobic and strength-conditioning exercises that are appropriate during pregnancy [44].

Women were encouraged to set achievable goals for dietary and physical activity change, supported to make these lifestyle changes, and asked to self-monitor their progress through the use of a workbook. Women were encouraged to identify potential barriers to implementation of their dietary goals. Using these perceived barriers, women were assisted to problem-solve, and to develop individualised strategies to facilitate their successful implementation.

This information was reinforced during subsequent inputs provided by the research dietician (at 28 weeks’ gestation) and trained research assistants (via telephone call at 22, 24, and 32 weeks’ gestation and a face-face visit at 36 weeks’ gestation).

#### Walking group (nested randomised controlled trial)

Women within the Lifestyle Advice group who had been further randomised to the Walking group were invited to participate in a targeted, supervised walking group, in addition to receiving the written and verbal information provided to all women assigned to the Lifestyle Advice group, as described above. The Walking group was designed to enable women to increase their level of physical activity through a simple form of exercise that could in future be performed without supervision, and that did not present a health risk to the pregnant woman or her unborn infant. Women were encouraged to participate three times per week, and to attend with a support person, under the guidance of a trained researcher.

There were three outdoor walking locations in the Adelaide metropolitan area, and two indoor walking locations in shopping centres. Indoor treadmill walking was provided at no cost as an alternative when the weather was not suitable for outdoor walking. Each walking session was designed to cover a distance of approximately 4.2 km, and was of 40 minutes duration (including 5 minutes of general stretching and warming up, followed by 5 minutes of cooling down time at the end of each session). The intensity of walking was moderate, consistent with recommendations in pregnancy [45]. During the walking session, women were provided with the opportunity to discuss the importance and effects of physical activity during pregnancy, including the optimal amount and intensity of home exercise.

#### Standard Care group

Women randomised to receive Standard Care continued their pregnancy care according to local hospital guidelines, which did not include routine provision of advice related to diet, exercise, or gestational weight gain.

### Study endpoints: maternal diet

The pre-specified endpoints related to maternal dietary intake were

- Food groups
- Macronutrient intake
- Micronutrient intake
- Healthy Eating Index (HEI)
- Dietary Glycaemic Load and Index

All women were asked to complete the Harvard Semi-quantitative Food Frequency questionnaire (the Willett questionnaire) at the time of study entry, at 28 and 36 weeks’ gestational age, and at 4 months postpartum. The Willett questionnaire was developed in 1985 in the USA to measure the daily intake of nutrients from 126 food items, with an indication of standard portion size, divided into seven food groups [46], and has been validated for use during pregnancy [47], and in an Australian pregnancy setting [48]. Questions were asked about the relative frequency of consumption of specific food items, use of supplements, cooking methods used and addition of sugar to foods. An open-ended question allowed record of consumption of other foods, which were then categorised by the study investigators. Daily nutrient intakes were estimated by multiplying frequency responses by the nutrient compositions of the specified portion size of each food item according to Australian food composition tables [49], reflecting standard food fortification with both folate and iodine. To assess adherence to dietary recommendations, food and drink consumption was grouped into food groups as defined by the Australian Guide to Healthy Eating [43]. Foods that did not fit into the five core food groups were classed as ‘non-core foods’ being higher in fat, sugar and salt, and providing minimal nutrients [43].

For the questionnaire completed at study entry, women were asked to indicate how often on average they had consumed the amount of food during the past year. For the questionnaires completed at 28 and 36 weeks and postpartum, women were asked to indicate how often on average they had consumed the amount of food since the previous questionnaire was completed.

Micronutrient values were obtained from the Willett questionnaire and analysed as mean intakes, utilising the Food Works Nutrient Analysis Software Package (FoodWorks, v.7 Professional; Xyris Software 2012; Australia) incorporating Australian food composition tables.

The 2005 HEI was used as an index of diet quality [50], consisting of 12 components, with a maximum score of 100. Total fruit (including 100% juice), whole fruits, total vegetables, dark-green and orange vegetables and legumes, total grains and whole grains categories have a score out of 5; milk, meat and beans, oils, saturated fat and sodium have scores out of 10; and calories from solid fats, alcoholic beverages and added sugars (SoFAAS) have a score out of 20. A HEI score above 80 is considered good, a score between 50 and 80 needs improvement, and scores below 50 are considered poor. The HEI has been validated for use in a pregnant population [51].

GI values were obtained from the Willett questionnaire and analysed as mean intakes, utilising the Food Works Nutrient Analysis Software Package (as above), incorporating Australian food composition tables and published glycaemic index values. Dietary glycaemic index was determined as the sum of the glycaemic index for all carbohydrates consumed in the diet, with a proportional weighting to account for the relative contribution of each food.

### Study endpoints: maternal physical activity

Women completed the Short Questionnaire to Assess Health-enhancing Physical Activity (SQUASH) [52] at trial entry, 28 and 36 weeks’ gestation, and 4 months postpartum. The questionnaire comprises 11 questions evaluating time spent on different types of physical activity (including commuting, leisure, household and incidental, and work-related activities), has been validated against accelerometer data [52], and has been used during pregnancy [53] and the post-partum period [54]. An activity-specific intensity code from the Compendium of Physical Activities [55] was assigned to each reported activity, and a corresponding estimate of intensity in metabolic equivalent task units (METs) was determined, where 1 MET is equal to the energy expended during quiet sitting [55]. The number of minutes spent in each reported activity was multiplied by its MET intensity, and summed to calculate total daily energy expenditure. Because MET is a measure of intensity and rate of physical activity, the concept of the MET-minute was used to quantify the total amount of physical activity in a comparable way between individuals and across activities [55]. As the SQUASH questionnaire reports physical activity during an average week, MET-minutes per week (METs/week) were calculated as duration (min) × frequency (days/week) of MET intensity.

### Analysis and reporting of results

Analyses were performed on an intention-to-treat basis. Women were included in the analysis if they returned one or more ‘valid’ questionnaires, and did not withdraw consent to use their data or did not have a miscarriage, termination of pregnancy, or stillbirth. Diet questionnaires were considered invalid if over 25% of responses were missing or if total energy intake was unrealistic (<4,500 kJ or >20,000 kJ) [56]. Physical activity questionnaires were considered invalid if the total hours of activity reported per week exceeded the number of hours in a week.

Outcomes were analysed using linear mixed effects models including treatment group, time, and their interaction, with adjustment made for the stratification variables centre, parity and BMI as fixed effects. Outcomes measured on different subjects were assumed to be independent, but outcomes measured on the same subject across the four time points were allowed to be correlated by specifying an unstructured covariance matrix for the error term. Baseline differences were allowed between treatment groups, as questionnaires were completed after women had been notified of their treatment group allocation, which may have influenced their responses. When the treatment × time interaction was significant, *post hoc* tests were performed to assess the effect of treatment group at each time point. When the interaction was not significant, it was removed from the model, and the main effect of treatment group was estimated. Exploratory analyses were also conducted to assess whether the effect of treatment varied by BMI category (overweight versus obese), by including an interaction between treatment group, time and BMI category (where the effect of treatment varied over time) or an interaction between treatment group and BMI category. Statistical significance was assessed at the two-sided *P* < 0.05 level, and no adjustment was made for multiple comparisons. All analyses were performed using SAS software (v9.3; SAS Inc., Cary, NC, USA).

### Sample size

The sample size of 2,180 women was pre-determined based on the primary outcome of the trial (large for gestational age infant) as reported previously [41].

## Results

**Figure 1:**
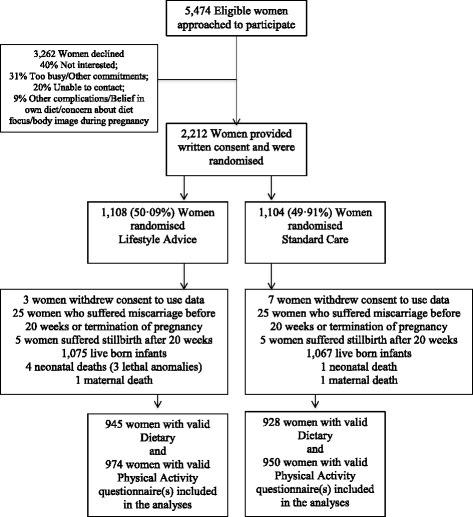
**Flow of participants through the trial.**

Between June 2008 and December 2011, we recruited and randomised 2,212 women, with 1,108 allocated to receive Lifestyle Advice and 1,104 Standard Care. There were 2,142 women (1,075 Lifestyle Advice; 1,067 Standard Care) available for inclusion in the analyses, after excluding women who withdrew consent to use their data (10 women) or had a miscarriage, termination of pregnancy or stillbirth (60 women) [41]. At least one valid dietary questionnaire [56] was received from 945 women (87.9%) in the Lifestyle Advice group, and 928 women (87.0%) in the Standard Care group. At least one valid physical activity questionnaire was received from 974 women (90.6%) in the Lifestyle Advice group, and 950 women (89.0%) in the Standard Care group who were included in the analyses (Figure 1). Baseline characteristics of the women who completed any questionnaires were similar between treatment groups (Table 1) and to the full randomised groups [41]. Number of questionnaires returned at each time point (study entry, 28 and 36 weeks’ gestational age, and at 4 months postpartum) are shown in Additional file 1: Table S1.

**Table 1:** **Demographic and clinical characteristics at trial entry**

**Characteristic**	**Lifestyle advice (n = 974)**	**Standard care (n = 950)**	**Total (n = 1924)**
Maternal age, years^a^	29.4 ± 5.4	29.6 ± 5.4	29.5 ± 5.4
Gestational age at entry, weeks^b^	14.3 (12.0 to 17.0)	14.3 (12.0 to 17.1)	14.3 (12.0 to 17.1)
Body mass index category, n (%)			
25.0 to 29.9	410 (42.1)	421 (44.3)	831 (43.2)
30.0 to 34.9	282 (29.0)	268 (28.2)	550 (28.6)
35.0 to 39.9	180 (18.5)	155 (16.3)	335 (17.4)
≥40.0	102 (10.5)	106 (11.2)	208 (10.8)
Public patient, n (%)	954 (97.9)	927 (97.6)	1881 (97.8)
Caucasian, n (%)	883 (90.7)	866 (91.2)	1749 (90.9)
Smoker, n (%)	124 (12.7)	97 (10.2)	221 (11.5)
Nulliparous, n (%)	410 (42.1)	385 (40.5)	795 (41.3)
Index of Socio-economic Disadvantage, n(%)^c^			
Unknown	2 (0.2)	1 (0.1)	3 (0.2)
Quintile 1 (most disadvantaged)	296 (30.4)	274 (28.8)	570 (29.6)
Quintile 2	231 (23.7)	236 (24.8)	467 (24.3)
Quintile 3	157 (16.1)	143 (15.1)	300 (15.6)
Quintile 4	138 (14.2)	151 (15.9)	289 (15.0)
Quintile 5 (least disadvantaged)	150 (15.4)	145 (15.3)	295 (15.3)

^a^Mean ± standard deviation.

^b^Median (interquartile range).

^c^Index of Socio-economic - disadvantage as measured by SEIFA (socioeconomic indexes for areas [57]).

### Macronutrient consumption and food groups

There was no statistically significant difference identified in the average daily energy consumption between women receiving Lifestyle Advice and women receiving Standard Care (Table 2). However, women randomised to receive Lifestyle Advice demonstrated a significant increase in the number of servings per day of fruit overall and vegetables after trial entry, compared with women receiving Standard Care. Additionally, women receiving Lifestyle Advice increased their consumption of dietary fibre, while significantly reducing the percentage of their energy intake derived from saturated fats overall compared with women receiving Standard Care. There was no evidence to suggest that the intervention effect was modified by maternal BMI category (data not shown).

**Table 2:** **Food group and macronutrient consumption: between treatment group comparison** ^**a**^

**Outcome**	**Time point**	**Lifestyle advice (n = 945)** ^**b**^	**Standard care (n = 928)** ^**b**^	**Adjusted treatment × time interaction** ***P*** **-value** ^**c**^	**Adjusted treatment effect**	
					***P***	**95% ** **CI**
Total energy, kJ^d^	Trial entry	8678.89 (2690.39)	8501.40 (2565.19)	0.99	0.09	178.60 (−26.56 to 383.77)
	28 weeks	8772.33 (2545.60)	8675.26 (2792.00)			
	36 weeks	8667.72 (2585.28)	8568.86 (2751.49)			
	4 months	8763.08 (2694.71)	8628.89 (2686.06)			
Breads and cereals, servings/day^d^	Trial entry	2.63 (1.45)	2.61 (1.43)	0.82	0.27	0.06 (−0.04 to 0.16)
	28 weeks	2.65 (1.34)	2.59 (1.42)			
	36 weeks	2.66 (1.33)	2.59 (1.42)			
	4 months	2.58 (1.39)	2.51 (1.37)			
Dairy, servings/day	Trial entry	2.05 (1.34)	2.21 (1.52)	0.002	0.02	−0.17 (−0.30 to −0.03)
	28 weeks	2.30 (1.24)	2.24 (1.39)		0.09	0.12 (−0.02 to 0.25)
	36 weeks	2.42 (1.42)	2.33 (1.40)		0.24	0.09 (−0.06 to 0.24)
	4 months	2.28 (1.36)	2.24 (1.42)		0.56	0.05 (−0.11 to 0.21)
Fruit, servings/day^d^	Trial entry	2.67 (2.07)	2.48 (1.63)	0.44	0.002	0.21 (0.08 to 0.35)
	28 weeks	2.70 (1.62)	2.43 (1.74)			
	36 weeks	2.56 (1.47)	2.34 (1.67)			
	4 months	2.24 (1.64)	2.11 (1.56)			
Meat and legumes, servings/day^d^	Trial entry	2.14 (1.03)	2.10 (0.94)	0.67	0.14	0.06 (−0.02 to 0.14)
	28 weeks	2.17 (1.08)	2.10 (0.97)			
	36 weeks	2.14 (0.91)	2.08 (1.05)			
	4 months	2.30 (0.95)	2.29 (1.08)			
Vegetables, servings/day	Trial entry	4.96 (2.64)	4.82 (2.57)	0.03	0.30	0.13 (−0.12 to 0.38)
	28 weeks	5.16 (2.68)	4.71 (2.37)		0.0002	0.47 (0.22 to 0.72)
	36 weeks	4.87 (2.54)	4.51 (2.65)		0.003	0.40 (0.13 to 0.67)
	4 months	5.63 (3.12)	5.24 (2.62)		0.003	0.50 (0.17 to 0.82)
Non-core group foods, servings/day	Trial entry	7.51 (3.81)	7.27 (3.61)	0.01	0.10	0.29 (−0.06 to 0.65)
	28 weeks	6.84 (3.24)	7.14 (3.41)		0.17	−0.24 (−0.58 to 0.10)
	36 weeks	6.86 (3.54)	6.96 (3.24)		0.72	−0.06 (−0.41 to 0.28)
	4 months	8.15 (3.69)	7.95 (3.76)		0.41	0.17 (−0.24 to 0.59)
Alcohol, g^d^	Trial entry	2.25 (5.21)	2.48 (6.47)	0.59	0.20	0.07 (−0.03 to 0.17)
	28 weeks	0.34 (1.35)	0.25 (0.86)			
	36 weeks	0.31 (1.42)	0.27 (1.06)			
	4 months	2.27 (4.04)	2.18 (4.92)			
Dietary fibre, g^d^	Trial entry	32.39 (12.91)	31.64 (12.43)	0.16	0.002	1.55 (0.55 to 2.56)
	28 weeks	33.75 (12.39)	31.88 (12.42)			
	36 weeks	32.78 (11.80)	30.93 (12.89)			
	4 months	34.09 (13.61)	32.50 (12.68)			
Carbohydrates, g^d^	Trial entry	255.27 (90.54)	246.38 (83.50)	0.69	0.06	6.55 (−0.19 to 13.29)
	28 weeks	258.11 (82.97)	255.89 (93.44)			
	36 weeks	253.61 (85.76)	251.43 (90.66)			
	4 months	247.86 (89.54)	243.25 (88.71)			
Percentage energy from carbohydrates^d^	Trial entry	46.85 (5.97)	46.22 (5.59)	0.23	0.39	0.19 (−0.24 to 0.62)
	28 weeks	46.96 (5.33)	46.89 (5.30)			
	36 weeks	46.61 (5.44)	46.68 (5.81)			
	4 months	44.89 (5.91)	44.76 (6.18)			
Protein, g^d^	Trial entry	97.59 (30.65)	97.80 (30.58)	0.07	0.14	1.82 (−0.57 to 4.22)
	28 weeks	100.07 (32.16)	97.56 (30.59)			
	36 weeks	100.13 (30.22)	97.10 (32.18)			
	4 months	102.67 (31.15)	101.24 (30.08)			
Percentage energy from protein	Trial entry	22.72 (3.83)	23.22 (3.88)	0.0001	0.008	−0.49 (−0.86 to −0.13)
	28 weeks	22.98 (3.74)	22.82 (3.66)		0.11	0.31 (−0.07 to 0.68)
	36 weeks	23.35 (3.85)	22.98 (4.04)		0.11	0.33 − 0.08 to 0.75)
	4 months	23.80 (4.25)	23.87 (4.26)		0.80	−0.06 (−0.55 to 0.42)
Total Fat, g^d^	Trial entry	65.28 (23.60)	64.33 (22.27)	0.83	0.48	0.64 (−1.14 to 2.41)
	28 weeks	66.54 (22.50)	66.70 (23.62)			
	36 weeks	65.98 (22.14)	66.24 (23.70)			
	4 months	68.16 (23.68)	67.72 (23.99)			
Percentage energy from total fat^d^	Trial entry	27.77 (4.51)	27.97 (4.20)	0.42	0.06	−0.31 (−0.64 to 0.02)
	28 weeks	28.02 (4.19)	28.45 (4.19)			
	36 weeks	28.13 (4.23)	28.59 (4.36)			
	4 months	28.76 (4.31)	29.00 (4.39)			
Saturated fat, g^d^	Trial entry	26.47 (10.98)	26.08 (10.01)	0.50	0.71	0.15 (−0.64 to 0.94)
	28 weeks	26.98 (9.87)	27.37 (10.30)			
	36 weeks	27.10 (10.20)	27.48 (10.62)			
	4 months	27.49 (10.51)	27.25 (10.29)			
Percentage energy from saturated fat^d^	Trial entry	11.23 (2.50)	11.34 (2.38)	0.09	0.04	−0.20 (−0.38 to −0.01)
	28 weeks	11.37 (2.33)	11.70 (2.41)			
	36 weeks	11.52 (2.41)	11.85 (2.47)			
	4 months	11.59 (2.43)	11.68 (2.47)			
Monounsaturated fat, g^d^	Trial entry	22.60 (8.56)	22.28 (8.13)	0.75	0.62	0.16 (−0.48 to 0.81)
	28 weeks	22.99 (8.27)	23.12 (8.70)			
	36 weeks	22.72 (7.96)	22.81 (8.72)			
	4 months	23.69 (8.72)	23.65 (9.12)			
Polyunsaturated fat, g^d^	Trial entry	9.09 (3.50)	8.95 (3.59)	0.90	0.23	0.17 (−0.11 to 0.45)
	28 weeks	9.31 (3.67)	9.13 (3.81)			
	36 weeks	9.06 (3.33)	8.89 (3.71)			
	4 months	9.48 (3.71)	9.43 (4.16)			

^a^Values are mean ± SD and treatment effects are differences in means (with 95% confidence interval and *P* -value) by time point estimated from a linear mixed effects model including treatment, time and treatment × time, adjusted for centre, parity and BMI.

^b^Includes women who had a live birth, and who answered one or more questionnaires; excludes questionnaires with >25% missing responses, or where there was an unrealistic energy intake reported (<4,500 or >20,000 kJ).

^c^Where the treatment × time interaction was not statistically significant, it was dropped from the model.

^d^Values are mean ± SD and treatment effects are differences in means (with 95% confidence interval and *P*-value) across all time points estimated from a linear mixed effects model including treatment and time, adjusted for centre, parity and BMI.

### Micronutrient consumption

Women randomised to the Lifestyle Advice group demonstrated improvements in their dietary micronutrient intake. During pregnancy, women in the Lifestyle Advice group reported greater intake of calcium, potassium and vitamin B_2_, but this was not maintained postpartum (Table 3). These women also increased their consumption of vitamin A, vitamin C and folate overall, compared with women in the Standard Care group (Table 3). No significant changes were observed for other micronutrients. There was some evidence to suggest that the effect of the intervention on iodine, vitamin E and folate intake was modified by maternal BMI category (interaction *P* < 0.05 in all cases), with overall intake significantly increased in obese but not overweight women randomised to the Lifestyle Advice group (data not shown).

**Table 3:** **Dietary micronutrient consumption: between treatment group comparison** ^**a**^

**Outcome**	**Time point**	**Lifestyle advice (n = 945)** ^**b**^	**Standard care (n = 928)** ^**b**^	**Adjusted treatment × time interaction** ***P*** **-value** ^**c**^	**Adjusted treatment effect**	
					***P***	**95% CI**
Caffeine, mg^d^	Trial entry	142.59 (156.88)	136.59 (156.36)	0.57	0.57	3.68 (−8.90 to 16.26)
	28 weeks	134.63 (147.21)	132.76 (147.04)			
	36 weeks	129.61 (141.89)	128.55 (140.66)			
	4 months	202.78 (191.12)	191.83 (185.43)			
Sodium, mg^d^	Trial entry	2713.21 (1080.73)	2644.16 (1102.75)	0.86	0.10	70.55 (−13.71 to 154.81)
	28 weeks	2684.04 (1038.11)	2651.08 (1048.43)			
	36 weeks	2704.91 (1059.82)	2620.33 (1045.35)			
	4 months	2822.92 (1164.27)	2759.30 (1092.84)			
Calcium, mg	Trial entry	930.81 (388.80)	963.43 (429.48)	0.007	0.11	−32.10 (−71.25 to 7.04)
	28 weeks	1009.34 (366.54)	984.56 (400.48)		0.04	40.73 (1.57 to 79.88)
	36 weeks	1031.14 (421.66)	1003.76 (404.85)		0.15	31.99 (−11.04 to 75.02)
	4 months	1007.13 (415.11)	990.64 (416.75)		0.37	21.26 (−25.14 to 67.67)
Iron, mg^d^	Trial entry	13.79 (4.79)	13.57 (4.61)	0.83	0.08	0.33 (−0.04 to 0.70)
	28 weeks	14.21 (4.56)	13.94 (4.92)			
	36 weeks	14.03 (4.48)	13.69 (5.04)			
	4 months	14.51 (4.79)	14.08 (4.77)			
Zinc, mg^d^	Trial entry	11.71 (3.69)	11.71 (3.56)	0.08	0.11	0.23 (−0.06 to 0.52)
	28 weeks	12.14 (3.70)	11.82 (3.70)			
	36 weeks	12.12 (3.68)	11.75 (3.90)			
	4 months	12.36 (3.74)	12.12 (3.62)			
Magnesium, mg^d^	Trial entry	344.03 (116.89)	342.67 (116.44)	0.11	0.06	9.07 (−0.20 to 18.33)
	28 weeks	356.11 (110.62)	344.98 (119.15)			
	36 weeks	353.11 (112.18)	340.31 (117.27)			
	4 months	364.02 (119.85)	354.13 (118.78)			
Phosphorus, mg^d^	Trial entry	1586.85 (497.80)	1595.95 (515.48)	0.08	0.16	28.14 (−11.27 to 67.56)
	28 weeks	1657.50 (493.47)	1618.65 (509.91)			
	36 weeks	1671.51 (516.33)	1626.25 (518.62)			
	4 months	1689.35 (528.98)	1655.95 (514.58)			
Potassium, mg	Trial entry	3591.78 (1220.73)	3570.31 (1169.87)	0.05	0.69	23.15 (−90.33 to 136.63)
	28 weeks	3723.05 (1129.40)	3586.57 (1223.47)		0.004	173.10 (54.97 to 291.23)
	36 weeks	3674.33 (1131.83)	3541.08 (1185.20)		0.01	158.03 (38.14 to 277.92)
	4 months	3781.35 (1271.44)	3657.89 (1165.91)		0.06	127.88 (−4.96 to 260.72)
Iodine, μg^d^	Trial entry	204.05 (108.94)	205.81 (105.00)	0.37	0.38	3.54 (−4.36 to 11.44)
	28 weeks	210.93 (97.92)	206.26 (96.36)			
	36 weeks	215.94 (101.27)	209.86 (99.91)			
	4 months	214.81 (109.29)	212.34 (102.24)			
Vitamin A Active Equivalent, μg^d^	Trial entry	1475.35 (974.00)	1404.15 (752.52)	0.16	0.003	110.40 (36.48 to 184.32)
	28 weeks	1605.54 (1323.79)	1424.89 (890.08)			
	36 weeks	1462.83 (821.57)	1363.63 (877.70)			
	4 months	1658.00 (1077.44)	1539.68 (852.02)			
Retinol, μg^d^	Trial entry	366.23 (558.09)	336.37 (311.97)	0.34	0.33	18.56 (−18.64 to 55.75)
	28 weeks	422.55 (1087.70)	372.54 (607.22)			
	36 weeks	349.30 (400.65)	369.78 (508.35)			
	4 months	376.11 (626.42)	371.01 (464.27)			
Vitamin B_1_ (thiamine), mg^d^	Trial entry	1.55 (0.57)	1.52 (0.56)	0.50	0.07	0.04 (−0.00 to 0.08)
	28 weeks	1.61 (0.55)	1.57 (0.61)			
	36 weeks	1.62 (0.55)	1.56 (0.62)			
	4 months	1.58 (0.57)	1.54 (0.56)			
Vitamin B_2_ (riboflavin), mg	Trial entry	2.14 (0.84)	2.19 (0.87)	0.02	0.22	−0.05 (−0.13 to 0.03)
	28 weeks	2.33 (0.79)	2.28 (0.89)		0.05	0.08 (−0.00 to 0.17)
	36 weeks	2.37 (0.86)	2.33 (0.87)		0.23	0.06 (−0.03 to 0.15)
	4 months	2.34 (0.87)	2.29 (0.85)		0.24	0.06 (−0.04 to 0.15)
Niacin, mg^d^	Trial entry	22.60 (7.43)	22.49 (7.24)	0.32	0.09	0.49 (−0.08 to 1.07)
	28 weeks	23.02 (7.61)	22.57 (7.47)			
	36 weeks	23.04 (7.05)	22.35 (7.75)			
	4 months	23.89 (7.47)	23.25 (7.08)			
Vitamin C, mg^d^	Trial entry	165.11 (117.86)	152.24 (90.04)	0.50	0.02	8.87 (1.40 to 16.34)
	28 weeks	158.26 (91.27)	147.34 (100.64)			
	36 weeks	149.35 (87.94)	142.60 (99.13)			
	4 months	139.11 (89.17)	133.80 (77.94)			
Vitamin E, mg^d^	Trial entry	7.37 (2.90)	7.35 (2.91)	0.71	0.17	0.16 (−0.07 to 0.38)
	28 weeks	7.58 (2.77)	7.39 (2.97)			
	36 weeks	7.40 (2.71)	7.23 (2.85)			
	4 months	7.94 (3.10)	7.74 (3.14)			
Folate, μg^d^	Trial entry	529.62 (206.87)	520.74 (210.02)	0.49	0.03	17.49 (1.26 to 33.71)
	28 weeks	545.59 (203.52)	528.35 (212.15)			
	36 weeks	540.67 (196.49)	522.05 (213.62)			
	4 months	544.99 (206.99)	528.74 (201.97)			
Folate food, μg^d^	Trial entry	401.20 (161.35)	395.15 (160.90)	0.24	0.02	15.13 (2.14 to 28.12)
	28 weeks	410.82 (161.38)	394.97 (161.04)			
	36 weeks	402.80 (150.79)	388.02 (161.70)			
	4 months	420.96 (164.11)	406.71 (156.54)			

^a^Values are mean ± SD and treatment effects are differences in means (with 95% confidence interval and *P*-value) by time point estimated from a linear mixed effects model including treatment, time and treatment × time, adjusted for centre, parity and body mass index.

^b^Includes women who had a live birth, and who answered one or more questionnaires; excludes questionnaires with >25% missing responses, or where there was an unrealistic energy intake reported (<4,500 or >20,000 kJ).

^c^Where the treatment × time interaction was not statistically significant, it was dropped from the model.

^d^Values are mean ± SD and treatment effects are differences in means (with 95% confidence interval and *P*-value) across all time points estimated from a linear mixed effects model including treatment and time, adjusted for centre, parity and BMI.

### Healthy Eating Index

Women randomised to receive Lifestyle Advice demonstrated significant improvement in their diet quality as measured by the HEI, at both 28 and 36 weeks’ gestation, compared with women receiving Standard Care (Table 4). Specifically, women receiving Lifestyle Advice significantly increased their consumption of total fruit, whole fruit, and dark-green and orange vegetables and legumes, compared with women receiving Standard Care. With the exception of improved consumption of dark-green and orange vegetables and legumes, these changes were not maintained at four months postpartum. The intervention was not associated with changes in consumption of grains, meat and beans, oils, sodium, or caloric intake from SoFAAS. There was no evidence of modification of the intervention effect by maternal BMI category (data not shown).

**Table 4:** **Healthy eating index: between treatment group comparison** ^**a**^

**Outcome**	**Time point**	**Lifestyle advice (n = 945)** ^**b**^	**Standard care (n = 928)** ^**b**^	**Adjusted treatment × time interaction** ***P*** **-value** ^**c**^	**Adjusted treatment effect**	
					***P***	**95% CI**
HEI, range 0 to 100	Trial entry	72.11 (7.71)	72.80 (7.07)	<0.0001	0.06	−0.67 (−1.37 to 0.03)
	28 weeks	73.35 (6.62)	71.86 (7.01)		<0.0001	1.58 (0.89 to 2.27)
	36 weeks	72.95 (6.82)	71.17 (7.69)		<0.0001	1.77 (1.01 to 2.53)
	4 months	72.83 (7.56)	72.15 (7.47)		0.41	0.35 (−0.48 to 1.18)
Total fruit, range 0 to 5	Trial entry	4.50 (1.02)	4.48 (1.03)	0.003	0.567	0.03 (−0.07 to 0.13)
	28 weeks	4.63 (0.89)	4.42 (1.11)		0.0001	0.20 (0.10 to 0.30)
	36 weeks	4.58 (0.97)	4.34 (1.18)		<0.0001	0.24 (0.13 to 0.35)
	4 months	4.27 (1.23)	4.11 (1.33)		0.07	0.14 (−0.01 to 0.28)
Whole fruit, range 0 to 5	Trial entry	4.56 (1.09)	4.57 (1.05)	0.0002	0.77	−0.01 (−0.12 to 0.09)
	28 weeks	4.68 (0.94)	4.50 (1.15)		0.0003	0.19 (0.09 to 0.30)
	36 weeks	4.64 (1.03)	4.42 (1.24)		<0.0001	0.24 (0.12 to 0.35)
	4 months	4.43 (1.18)	4.35 (1.24)		0.30	0.07 (−0.06 to 0.21)
Total vegetables, range 0 to 5^d^	Trial entry	4.86 (0.58)	4.88 (0.50)	0.06	0.12	0.03 (−0.01 to 0.06)
	28 weeks	4.91 (0.44)	4.88 (0.48)			
	36 weeks	4.88 (0.49)	4.81 (0.62)			
	4 months	4.94 (0.31)	4.89 (0.45)			
Dark-green and orange Vegetables and legumes, range 0 to 5^d^	Trial entry	4.76 (0.76)	4.72 (0.84)	0.12	0.0006	0.10 (0.04 to 0.16)
	28 weeks	4.82 (0.64)	4.73 (0.78)			
	36 weeks	4.76 (0.77)	4.64 (0.93)			
	4 months	4.89 (0.46)	4.78 (0.75)			
Total grains, range 0 to 5^d^	Trial entry	3.88 (0.93)	3.89 (0.91)	0.24	0.55	−0.02 (−0.09 to 0.05)
	28 weeks	3.88 (0.91)	3.95 (0.88)			
	36 weeks	3.93 (0.91)	3.92 (0.93)			
	4 months	3.80 (1.00)	3.78 (1.04)			
Whole grains, range 0 to 5^d^	Trial entry	0.71 (0.88)	0.71 (0.83)	0.23	0.14	0.05 (−0.02 to 0.11)
	28 weeks	0.74 (0.86)	0.67 (0.83)			
	36 weeks	0.82 (0.90)	0.71 (0.91)			
	4 months	0.81 (0.87)	0.78 (0.88)			
Milk, range 0 to 10	Trial entry	6.54 (2.80)	6.98 (2.82)	<0.0001	0.002	−0.42 (−0.69 to −0.16)
	28 weeks	7.37 (2.61)	7.15 (2.70)		0.04	0.29 (0.01 to 0.56)
	36 weeks	7.48 (2.49)	7.34 (2.69)		0.45	0.10 (−0.17 to 0.38)
	4 months	7.23 (2.70)	7.01 (2.69)		0.28	0.17 (−0.14 to 0.48)
Meat and beans, range 0 to 10^d^	Trial entry	9.59 (1.20)	9.61 (1.07)	0.24	0.67	0.02 (−0.06 to 0.10)
	28 weeks	9.59 (1.16)	9.55 (1.16)			
	36 weeks	9.60 (1.13)	9.46 (1.26)			
	4 months	9.74 (0.96)	9.74 (0.99)			
Oils, range 0 to 10^d^	Trial entry	5.48 (3.19)	5.45 (3.25)	0.24	0.15	0.18 (−0.07 to 0.42)
	28 weeks	5.50 (3.17)	5.27 (3.18)			
	36 weeks	5.42 (3.27)	4.99 (3.25)			
	4 months	5.69 (3.18)	5.46 (3.26)			
Saturated fat, range 0 to 10^d^	Trial entry	5.48 (3.00)	5.36 (2.96)	0.08	0.07	0.21 (−0.02 to 0.44)
	28 weeks	5.31 (2.88)	4.91 (2.90)			
	36 weeks	5.04 (3.04)	4.70 (3.02)			
	4 months	5.01 (3.02)	4.97 (3.00)			
Sodium, range 0 to 10^d^	Trial entry	6.10 (2.43)	6.22 (2.43)	0.77	0.34	−0.09 (−0.27 to 0.10)
	28 weeks	6.28 (2.42)	6.27 (2.32)			
	36 weeks	6.10 (2.44)	6.28 (2.38)			
	4 months	5.80 (2.63)	5.91 (2.46)			
Calories from solid fats, alcohol and added sugar (SoFAAS), range 0 to 20^d^	Trial entry	15.64 (3.79)	15.94 (3.60)	0.150	0.56	−0.08 (−0.33 to 0.18)
	28 weeks	15.63 (3.24)	15.55 (3.45)			
	36 weeks	15.69 (3.20)	15.57 (3.51)			
	4 months	16.21 (3.33)	16.37 (3.22)			

^a^Values are mean ± SD and treatment effects are differences in means (with 95% confidence interval and *P*-value) by time point estimated from a linear mixed effects model including treatment, time and treatment × time, adjusted for centre, parity and BMI.

^b^Includes women who had a live birth, and who answered one or more questionnaires; excludes questionnaires with >25% missing responses, or where there was an unrealistic energy intake reported (<4,500 or >20,000 kJ).

^c^Where the treatment × time interaction was not statistically significant, it was dropped from the model.

^d^Values are mean ± SD and treatment effects are differences in means (with 95% confidence interval and *P*-value) across all time points estimated from a linear mixed effects model including treatment and time, adjusted for centre, parity and BMI.

### Glycaemic index and glycaemic load

There were no statistically significant differences identified in dietary glycaemic load or dietary glycaemic index between women receiving Lifestyle Advice and women receiving Standard Care (Table 5). There was also no evidence of modification of the intervention effect by maternal BMI category (data not shown).

**Table 5:** **Glycaemic load and glycaemic index: between treatment group comparison** ^**a**^

**Outcome**	**Time point**	**Lifestyle advice (n = 945)** ^**b**^	**Standard care (n = 928)** ^**b**^	**Adjusted treatment × time interaction** ***P*** **-value** ^**c**^	**Adjusted treatment effect**	
					***P***	**95% CI**
Glycaemic load	Trial entry	129.46 (47.84)	124.92 (45.07)	0.45	0.15	2.62 (−0.94 to 6.18)
	28 weeks	130.10 (44.55)	129.86 (50.67)			
	36 weeks	126.96 (44.90)	127.12 (48.60)			
	4 months	123.56 (47.92)	121.86 (47.71)			
Glycaemic index	Trial entry	50.57 (3.65)	50.51 (3.59)	0.17	0.10	−0.22 (−0.48 to 0.04)
	28 weeks	50.19 (3.36)	50.48 (3.45)			
	36 weeks	49.90 (3.47)	50.32 (3.65)			
	4 months	49.55 (3.92)	49.71 (4.17)			

^a^Values are mean ± SD and treatment effects are differences in means (with 95% confidence interval and *P*-value) across all time points estimated from a linear mixed effects model including treatment and time, adjusted for centre, parity and BMI.

^b^Includes women who had a live birth, and who answered one or more questionnaires; excludes questionnaires with >25% missing responses, or where there was an unrealistic energy intake reported (<4,500 or >20,000 kJ).

^c^Where the treatment × time interaction was not statistically significant, it was dropped from the model.

### Physical activity

Women randomised to receive Lifestyle Advice demonstrated a significant overall improvement in total physical activity, compared with women receiving Standard Care (Table 6). This finding was driven by an overall increase in household activity, with some evidence of an increase in leisure activity also, and represented an additional 15 to 20 minutes brisk walking on most days of the week. Commuting and work-related activities were similar between groups and at each time point for women who engaged in these activities (data not shown). No changes in treatment effects over time were identified for any types of activity (Table 6). There was some evidence to suggest that the effect of the intervention was modified by maternal BMI category for total physical activity (*P* = 0.043), with women who were obese demonstrating the greatest increase in total physical activity (adjusted difference in means 617.20 MET-minutes/week; 95% CI 238.48 to 995.92; *P* = 0.001).

**Table 6:** **Self-reported physical activity: between treatment group comparison** ^**a**^

**Outcome**	**Time point**	**Lifestyle advice (n = 974)** ^**b**^	**Standard care (n = 950)** ^**b**^	**Adjusted treatment × time interaction** ***P*** **-value** ^**c**^	**Adjusted treatment effect**	
					***P***	**95% CI**
Commuting activity^d^	Trial entry	286.15 (386.14)	245.40 (277.01)	0.56	0.55	11.83 (−26.75 to 50.42)
	28 weeks	234.95 (234.09)	219.74 (226.69)			
	36 weeks	212.05 (257.66)	228.88 (314.25)			
	4 months	309.87 (331.10)	330.50 (422.89)			
Leisure activity	Trial entry	1081.25 (1423.50)	1022.28 (1282.32)	0.22	0.06	79.33 (−2.09 to 160.75)
	28 weeks	1016.04 (1310.73)	862.28 (1092.86)			
	36 weeks	788.66 (961.96)	777.53 (900.00)			
	4 months	1281.07 (1303.30)	1163.67 (1249.04)			
Household activity	Trial entry	3290.48 (3139.01)	3148.30 (3093.46)	0.59	0.01	265.60 (61.36 to 469.84)
	28 weeks	3229.92 (3066.10)	2988.91 (2961.08)			
	36 weeks	3158.49 (2954.16)	2813.98 (2934.20)			
	4 months	4756.43 (3831.12)	4677.16 (3881.09)			
Work activity^d^	Trial entry	4697.83 (3093.41)	4405.76 (2818.43)	0.40	0.52	80.85 (−163.12 to 324.83)
	28 weeks	4326.30 (2707.53)	4279.65 (2660.85)			
	36 weeks	4032.73 (2484.61)	4059.23 (2593.77)			
	4 months	3041.02 (2505.86)	3204.85 (2706.89)			
Total activity	Trial entry	7587.63 (4573.52)	7259.93 (4145.34)	0.99	0.01	359.76 (74.87 to 644.65)
	28 Weeks	7010.32 (3950.39)	6742.48 (3836.85)			
	36 Weeks	5819.82 (3954.63)	5518.10 (3844.79)			
	4 Months	6530.19 (4336.80)	6317.00 (4498.15)			

^a^Values are mean ± SD and treatment effects are differences in means (with 95% confidence interval and *P*-value) across all time points estimated from a linear mixed effects model including treatment and time, adjusted for centre, parity and BMI.

^b^Includes women who had a live birth, and who answered one or more questionnaires; excludes questionnaires where the total hours of activity per week reported exceeded the number of hours in a week.

^c^Where the treatment × time interaction was not statistically significant, it was dropped from the model.

^d^Where women participated in these activities.

#### Nested randomised trial

**Figure 2:**
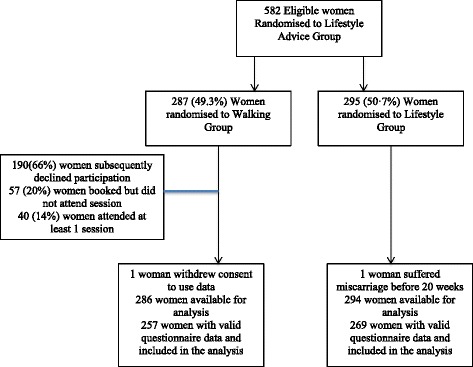
**Flow of participants through the nested randomised trial.**

During the specified time period, 582 eligible women from the Lifestyle Advice group were further randomised, 287 to the Walking group, and 295 to the Lifestyle group. Baseline characteristics of the women who participated in the nested randomised trial are included in Additional file 2: Table S2 and were similar between treatment groups. There were 580 women (286 Walking group; 294 Lifestyle group) available for inclusion in the analyses, after 1 woman had a miscarriage, and 1 woman withdrew consent to utilise her data. At least 1 valid physical activity questionnaire was received from 257 women in the Walking group (89.9%), and 269 women (91.5%) in the Lifestyle group (Figure 2). At trial entry, the characteristics of the women included in the analysis were similar between treatment groups (data not shown).

Of the women randomised to the Walking group, 190 (66%) subsequently declined to participate in the Walking group when contacted despite their initial consent, 57 (20%) booked but subsequently did not attend a walking session, and 40 (14%) attended at least one session. The median number of sessions attended was 2 (interquartile range 1 to 7). In women who participated in the Walking group, no serious adverse effects of exercise (including syncope, chest pain, shortness of breath, vaginal bleeding or miscarriage) were identified.

Women further randomised to the Walking group demonstrated no significant difference in total physical activity compared with women in the Lifestyle group (Table 7). Commuting, housework and work-related activities were similar between groups. There was some evidence to suggest that treatment effects varied over time for leisure activities (interaction *P* = 0.04), but no significant differences were identified between treatment groups at any time point in *post hoc* testing.

**Table 7:** **Self-reported physical activity of participants in nested randomised trial: between treatment group comparison** ^**a**^

**Outcome**	**Time point**	**Walking group (n = 257)** ^**b**^	**Information only (n = 269)** ^**b**^	**Adjusted treatment × time interaction** ***P*** **-value** ^**c**^	**Adjusted treatment effect**	
					***P***	**95% CI**
Commuting activity^d^	Trial entry	227.30 (281.27)	240.47 (243.97)	0.24	0.63	17.41 (−54.57 to 89.39)
	28 weeks	218.05 (176.55)	200.32 (287.47)			
	36 weeks	292.52 (366.32)	185.01 (178.98)			
	4 months	248.44 (263.20)	390.00 (512.82)			
Leisure activity^e^	Trial entry	1048.02 (1311.53)	877.42 (1146.34)	0.04	0.11	174.72 (−41.93 to 391.38)
	28 weeks	1048.30 (1248.43)	911.11 (1212.73)		0.36	108.50 (−125.45 to 342.44)
	36 weeks	796.16 (1011.25)	727.39 (810.12)		0.67	52.10 (−188.17 to 292.38)
	4 months	1178.16 (1071.98)	1374.64 (1411.67)		0.08	−236.04 (−503.44 to 31.37)
Household activity	Trial entry	3437.45 (3353.79)	2961.20 (2924.20)	0.19	0.16	294.44 (−114.88 to 703.76)
	28 weeks	3441.23 (3085.54)	2697.92 (2732.72)			
	36 weeks	3208.16 (3005.22)	2951.82 (3111.69)			
	4 months	4862.53 (4204.93)	4672.32 (3766.02)			
Work-related activity^d^	Trial entry	4440.89 (2743.25)	4888.99 (3130.39)	0.19	0.26	−259.57 (−710.22 to 191.08)
	28 weeks	4121.12 (2359.13)	4409.20 (2735.41)			
	36 weeks	3512.04 (1852.27)	4035.94 (2274.21)			
	4 months	3454.71 (2619.25)	3144.64 (2212.72)			
Total activity	Trial entry	7524.07 (4657.16)	7311.45 (4318.89)	0.75	0.40	247.31 (−332.35 to 826.98)
	28 weeks	7169.05 (3823.28)	6564.51 (3898.19)			
	36 weeks	5888.77 (3688.15)	5479.91 (4066.69)			
	4 months	6526.84 (4637.30)	6641.77 (4494.63)			

^a^Values are mean ± SD and treatment effects are differences in means (with 95% confidence interval and *P*-value) across all time points estimated from a linear mixed effects model including treatment and time, adjusted for centre, parity and BMI.

^b^Includes women who had a live birth, and who answered one or more questionnaires; excludes questionnaires where the total hours of activity per week reported exceeded the number of hours in a week.

^c^Where the treatment × time interaction was not statistically significant, it was dropped from the model.

^d^Where women participated in these activities.

^e^Values are mean ± SD and treatment effects are differences in means (with 95% confidence interval and P-value) by time point estimated from a linear mixed effects model including treatment, time and treatment × time, adjusted for centre, parity and BMI.

## Discussion

Our randomised trial is the largest reported to date evaluating specific dietary and physical activity effects of an antenatal lifestyle intervention for women who are overweight or obese during pregnancy, utilising robust methodology, and is the first to compare differing intensities of provision of support for increasing physical activity. The findings indicate that provision of an intervention during pregnancy is effective in improving maternal diet. Specifically, our data suggest that intake of fibre, saturated fat, fruits and vegetables, micronutrient intake, and overall maternal diet quality, as measured by the HEI, improved following the intervention, in the absence of significant changes in energy intake. Although some changes in diet quality and micronutrient consumption were evident during pregnancy, the improvements noted were often not maintained at 4 months postpartum, and there were no differences identified in maternal dietary glycaemic load or index. There was little evidence to suggest that the effect of the intervention differed between overweight and obese women.

The findings of the LIMIT Trial also indicate that provision of a lifestyle intervention during pregnancy was effective in increasing total physical activity, largely through increasing household activity, approximately equivalent to an additional 15 to 20 minutes brisk walking on most days of the week, an effect that was more pronounced among obese women. Although women were provided with access to a structured walking group in the nested component of the trial, attendance was poor, with women preferring a less supervised approach to physical activity.

We have previously reported an 18% relative risk reduction in infant birth weight above 4 kg following the provision of an antenatal intervention for pregnant women who are overweight or obese [41]. This observed effect on infant birth weight appears to have been mediated by changes in maternal diet quality and physical activity, despite the fact that maternal gestational weight gain [41], total energy intake during pregnancy, and dietary GL, did not differ significantly between the two randomised groups.

Detailed dietary changes following antenatal interventions to women who are overweight or obese have been poorly reported to date. To our knowledge, the current randomised trial is one of the few studies presenting detailed macronutrient, micronutrient, food group, dietary quality, and glycaemic index or glycaemic load information in an overweight or obese pregnant population. There is evidence from non-pregnant populations that subtle differences in HEI may be associated with improvements in health and reduced complications associated with type 2 diabetes [58]. However, other studies report larger differences in diet quality, in association with changes in blood pressure and other measures of cardiometabolic disease, including cholesterol [59,60]. We previously identified a decline in maternal HEI over the duration of pregnancy, which was maintained into the postpartum period, and was positively correlated with socioeconomic status [14]. While the currently reported HEI scores are higher than those previously derived from a far smaller subgroup of participants [14], the trend towards a decline in diet quality across pregnancy and into the postpartum period was also evident for all the women receiving standard care.

Overall, the existing literature supports the positive effect of lifestyle interventions during pregnancy for women who are overweight or obese in improving a range of measures of dietary intake [40,61-63], even in the absence of changes in gestational weight gain and overall energy intake [61,62]. Specific dietary modifications reported include reduced consumption of saturated fats [40,61-63] and increased consumption of protein [40,61,63]. The currently reported findings are consistent with maternal total energy intake observed in other studies [61,63], and although the percentage energy derived from fat was similar to that reported by Wolff and colleagues [63], it was lower than that reported by both Guelinckx [61] and Poston [40]. These differences may reflect variations in baseline characteristics, with the LIMIT cohort of women being predominantly of white ethnicity, and having lower mean BMI at trial entry.

The developmental over-nutrition hypothesis (also termed the Pedersen Hypothesis) was first proposed in 1954 [64] in an attempt to explain the relationship between maternal diabetes during pregnancy and fetal overgrowth, principally increased adiposity. Under this hypothesis, maternal hyperglycaemia is associated with increased placental transfer of glucose, resulting in fetal hyperglycaemia and increased insulin production, with the resultant effect being an increase in insulin-mediated fetal growth. More recently, the hypothesis has been expanded to recognise the potential metabolic impact of maternal obesity [65], which shares a similar metabolic milieu, characterised by insulin resistance, hyperglycaemia, hyperlipidaemia and a low-grade state of chronic inflammation, which in turn has been documented to influence the availability and transfer of nutrients to the developing fetus [65].

The findings of the LIMIT Trial did not identify differences in maternal dietary glycaemic index or glycaemic load following antenatal lifestyle intervention, which is in contrast to other reports in the literature [22,40]. The effects of maternal dietary glycaemic index on pregnancy outcomes generally, and measures of fetal and neonatal growth more specifically, are uncertain. Although some studies have identified an association between maternal consumption of a low glycaemic index diet and reduced gestational weight gain [22,23], lower infant birth weight [24] and reduced neonatal adiposity as measured by thigh circumference [66], these findings are not universal, with others reporting no evidence of effect on infant birth weight [23,25-27,40].

The contribution to fetal growth of other fuel substrates, including free fatty acids, triglycerides and amino acids, has also been recognised [64]. Fatty acids may be of importance as a fuel substrate for obese pregnant women, who have demonstrated increased reliance on lipid metabolism [67] in order to meet the energy requirements of pregnancy, which are only minimally accounted for by changes in dietary energy intake [68]. This is of particular relevance, given the currently reported findings of increased consumption of dietary fibre and reduced saturated fat intake, both of which could plausibly improve maternal insulin resistance [69] and contribute to our previously reported reduction in high infant birth weight following antenatal dietary and physical activity advice [41]. Furthermore, relatively modest changes in nutrient consumption and diet quality may directly affect clinical outcomes during pregnancy, particularly fetal and early infant growth. Reports in the literature indicate that maternal consumption of a diet high in polyunsaturated fatty acids is associated with a reduction in early childhood adiposity as measured by skinfold thickness [70], and is predictive of fat mass determined by dual energy X-ray absorptiometry at the ages of 4 and 6 years [71]. Together with our findings, these reports highlight the potential impact of relatively modest changes in maternal diet quality on *in utero* growth, birth weight and future childhood adiposity.

There is a lack of consensus as to the most appropriate tool to assess physical activity, both in the general population, and more specifically during pregnancy [72]. Although physical activity questionnaires are a cost-effective method of assessment, particularly for use in large-scale studies, concerns have been raised about them over-estimating activity [73]. The use of pedometers and accelerometers has been advocated as a more objective tool, although these are not without their limitations, including poor measurement of upper body movement and stationary exercise [72]. Furthermore, the two methods do not appear interchangeable, particularly for overweight and obese pregnant women, with poorly reported correlation in step counts for any individual [74].

The published literature consistently reports a reduction in physical activity as pregnancy advances [39], particularly among women who are overweight or obese [40] compared with lean women [38,39]. Reports suggest that domestic and childcare activities constitute up to 50% of total energy expenditure and activity during pregnancy [75,76], increasing to 65% in women who are obese [77]. It is therefore important that questionnaires include this activity category [75], particularly as other assessment measures, including both pedometers and accelerometers, are poor at identifying low-intensity activity [72].

A potential limitation of our trial is the reliance on self-reported questionnaire assessment of both dietary intake and physical activity. However, the purpose was to compare the effects of an antenatal intervention with standard care, and more detailed assessments of either dietary intake or physical activity were not considered feasible, given the sample size involved and the multiple time points assessed. A general concern with dietary and physical activity studies, particularly those relying on self-completed questionnaires, relates to the potential for recall bias, which may be differentially evident according to treatment group allocation. Even though women were asked about their dietary intake in the past 12 months at the time of trial entry, it is possible that the baseline assessment of both dietary intake and physical activity were influenced by knowledge of treatment group allocation. Although there was a fall-off in questionnaire response rates over pregnancy and the postpartum period, the proportion of women contributing data to the analyses was high. Furthermore, the women included in the analysis had similar baseline characteristics to, and can therefore be considered representative of, the complete randomised groups [41].

It is possible that women in the intervention group modified their self-reported dietary intake and physical activity following notification of the treatment group in order to provide ‘desirable’ answers and those that would be subsequently consistent with the content of the intervention sessions. However, if this were the case, we would have anticipated changes also to be reported in the consumption of refined carbohydrates and sugar-sweetened beverages, which was not observed. We observed an increase in reported physical activity among women in the intervention group, which was consistent across all time points, including trial entry. This may reflect variation in the timing of completion of the trial entry questionnaire, with women randomised to the Lifestyle Advice group potentially increasing their activity in the period between randomisation and questionnaire return (up to 10 days), prior to attendance for their first intervention session. It may also reflect a chance occurrence, particularly because, although dietary questionnaires were completed within the same time period, no baseline differences in dietary intake measures were observed between the two treatment groups. True differences in physical activity of this magnitude between treatment groups at trial entry are unlikely, given our large sample size and the degree of balance achieved for other baseline characteristics.

## Conclusions

To date, there has been a lack of detailed information from randomised trials outlining specific effects on maternal diet and physical activity, following an antenatal intervention for women who are overweight or obese. While our results indicate that provision of an antenatal dietary and lifestyle intervention is effective in improving maternal diet quality, food group, macronutrient and micronutrient intake, and physical activity during pregnancy, many of these improvements were not maintained at 4 months postpartum, highlighting the need for additional interventions during this important period of adjustment for women and their families.

We consider the observed changes in diet quality and physical activity during pregnancy, although modest, to be of clinical significance, given our reported findings of a reduced risk of infant birth weight above 4 kg for pregnant women who are overweight or obese [41], and such changes are likely to be far more achievable from a public health perspective than more restrictive approaches to limiting gestational weight gain. It will therefore be important to continue to follow up the infants born to women who participated in this trial to evaluate the longer-term health effects of the changes observed in maternal diet and physical activity achieved during pregnancy, particularly in relation to subsequent childhood obesity.

## Additional files

Additional file 1: Table S1. Questionnaire response by time point. Additional file 2: Table S2. Demographic and clinical characteristics at trial entry of participants in nested randomised trial.
