# Supplementary material for: The effects of antenatal dietary and lifestyle advice for women who are overweight or obese on maternal diet and physical activity: the LIMIT randomised trial
Source: BMC Med. 2014 Oct 13;12:161. doi: 10.1186/s12916-014-0161-y (PMC4194375; doi:10.1186/s12916-014-0161-y)
Supplement: Additional file 1: Table S1. — Questionnaire response by time point. [file 12916_2014_161_MOESM1_ESM.docx]

**Additional file 1: TABLE S1:** Questionnaire response by time point

|  | **Lifestyle Advice Group** | **Standard Care Group** |
| --- | --- | --- |
| **Trial Entry**  Dietary Questionnaire  Physical Activity Questionnaire | 878  901 | 877  885 |
| **28 weeks gestation**  Dietary Questionnaire  Physical Activity Questionnaire | 729  731 | 748  751 |
| **36 weeks gestation**  Dietary Questionnaire  Physical Activity Questionnaire | 679  699 | 684  689 |
| **4 months post-partum**  Dietary Questionnaire  Physical Activity Questionnaire | 576  587 | 601  626 |
