# Supplementary material for: The effects of antenatal dietary and lifestyle advice for women who are overweight or obese on maternal diet and physical activity: the LIMIT randomised trial
Source: BMC Med. 2014 Oct 13;12:161. doi: 10.1186/s12916-014-0161-y (PMC4194375; doi:10.1186/s12916-014-0161-y)
Supplement: Additional file 2: Table S2. — Demographic and clinical characteristics at trial entry of participants in nested randomised trial. [file 12916_2014_161_MOESM2_ESM.docx]

**Additional file 2: TABLE S2:** Demographic and clinical characteristics at trial entry of participants in nested randomised trial

| ***Characteristic*** | ***Walking Group (N=257)*** | ***Lifestyle Group (N=269)*** | ***Total (N=526)*** |  |
| --- | --- | --- | --- | --- |
| Maternal Age (Years)^*^ | 29.1 ( 5.3) | 29.6 ( 5.5) | 29.4 ( 5.4) |  |
| Gestational Age at Entry (Weeks)^+^ | 14.4 (12.3-17.3) | 14.7 (12.4-18.0) | 14.5 (12.3-17.6) |  |
| Body Mass Index Category^#^ |  |  |  |  |
| . BMI 25.0-29.9 | 113 (44.0) | 118 (43.9) | 231 (43.9) |  |
| . BMI 30.0-34.9 | 78 (30.4) | 74 (27.5) | 152 (28.9) |  |
| . BMI 35.0-39.9 | 43 (16.7) | 52 (19.3) | 95 (18.1) |  |
| . BMI >=40.0 | 23 (8.9) | 25 (9.3) | 48 (9.1) |  |
| Public Patient^#^ | 255 (99.2) | 267 (99.3) | 522 (99.2) |  |
| Caucasian^#^ | 239 (93.0) | 240 (89.2) | 479 (91.1) |  |
| Smoker^#^ | 36 (14.0) | 31 (11.5) | 67 (12.7) |  |
| Nulliparous^#^ | 104 (40.5) | 116 (43.1) | 220 (41.8) |  |
| Index of Socio-economic Disadvantage^ |  |  |  |  |
| . Unknown | 0 (0.0) | 1 (0.4) | 1 (0.2) |  |
| . Quintile 1 (Most Disadvantaged) | 75 (29.2) | 85 (31.6) | 160 (30.4) |  |
| . Quintile 2 | 64 (24.9) | 58 (21.6) | 122 (23.2) |  |
| . Quintile 3 | 44 (17.1) | 47 (17.5) | 91 (17.3) |  |
| . Quintile 4 | 30 (11.7) | 39 (14.5) | 69 (13.1) |  |
| . Quintile 5 (Least Disadvantaged) | 44 (17.1) | 39 (14.5) | 83 (15.8) |  |

* mean and standard deviation

^+^ median and interquartile range

^#^ number and %

^ Socio-economic index of disadvantage as measured by SEIFA
